# Sleep deprivation as a risk factor for cognitive decline among middle-aged adults

**DOI:** 10.6026/973206300213484

**Published:** 2025-10-31

**Authors:** Debadrit Biswas, Pooja Saithya Pillarisetti, Achyuth Prasad Jakka, Subash Kumar

**Affiliations:** 1 Department of Neurology, Ruby General Hospital, West Bengal, India; 2 Department of General Medicine, A.C.S.R Government Medical College Nellore, Andhra Pradesh, India; 3 General Medicine, Government Medical College, Anantapuramu, Andhra Pradesh, India; 4 Department of General Medicine, Madras Medical College, Chennai, India

**Keywords:** Sleep deprivation, cognitive decline, prospective study, middle-aged adults, memory impairment

## Abstract

The impact of chronic sleep deprivation on cognitive decline among 140 middle-aged adults aged 40-60 years over a 3-year period is of interest. Participants were assessed using standardized cognitive batteries and sleep tracking devices. Data revealed that individuals sleeping less than 6 hours per night had significantly greater declines in memory, executive function and attention. These associations remained significant after adjusting for confounders such as age, sex and comorbidities. Chronic insufficient sleep appears to be an independent predictor of accelerated cognitive decline.

## Background:

Cognitive decline in midlife is an important public health concern, as it may signify early stages of neurodegenerative processes and influence long-term brain health outcomes [1]. While numerous factors contribute to cognitive aging, sleep has emerged as a critical but often underestimated determinant. Sleep plays a vital role in memory consolidation, synaptic plasticity, toxin clearance (e.g., beta-amyloid) and overall brain restoration [2]. Chronic sleep deprivation is increasingly prevalent in modern society due to lifestyle, occupational demands and digital exposure, yet its longitudinal impact on cognitive functioning in otherwise healthy middle-aged adults remains underexplored. Most prior research has focused on older populations or short-term cognitive effects [3]. This study aims to prospectively evaluate whether persistent short sleep duration is independently associated with cognitive decline over time, particularly in domains such as memory, attention, processing speed and executive function. By targeting middle-aged individuals, this research seeks to identify an actionable, modifiable risk factor that could prevent or delay the onset of cognitive impairment [4]. Therefore, it is of interest to study on sleep deprivation as a risk factor for cognitive decline in middle-aged adults.

## Materials and Methods:

This prospective cohort study enrolled 140 cognitively healthy adults aged 40 to 60 years, recruited from urban primary care clinics and community wellness programs [5]. Individuals with a history of diagnosed sleep disorders, psychiatric illness, neurodegenerative disease, or on-going use of sedatives or cognitive enhancers were excluded. Baseline evaluations included detailed medical history, physical examination and standardized neuropsychological testing, including assessments of memory (Rey Auditory Verbal Learning Test), attention (Digit Span), executive function (Trail Making Test) and processing speed (Symbol Digit Modalities Test) [6]. Sleep patterns were objectively measured using actigraphy watches worn continuously for 14 days at baseline and then annually, capturing total sleep time, sleep latency and sleep efficiency [7]. Participants also completed subjective sleep quality questionnaires (Pittsburgh Sleep Quality Index). Based on total sleep duration, participants were categorized into three groups: short sleepers (<6 hours), normal sleepers (6-8 hours) and long sleepers (>8 hours) [8]. Cognitive testing was repeated annually for 3 years [9]. The primary outcome was change in global cognitive performance, while secondary outcomes included domain-specific decline. Mixed-effects regression models were used to examine the association between sleep duration and cognitive decline, adjusting for age, sex, BMI, education level, baseline cognition, physical activity and comorbid conditions (e.g., hypertension, diabetes) [10].

## Results:

Over the 3-year follow-up period, individuals with chronic sleep deprivation (<6 hours/night) demonstrated significantly greater declines in multiple cognitive domains compared to those with adequate or long sleep. At baseline, short sleepers presented with higher body mass index (BMI) and a greater burden of comorbidities, including hypertension and diabetes, despite similar distributions of age and sex across groups. They also reported markedly poorer subjective sleep quality. Longitudinally, short sleepers exhibited the steepest deterioration in global cognition, memory, executive function and working memory, with performance gaps widening steadily over time. Multivariate regression confirmed that insufficient sleep was an independent predictor of cognitive decline, even after accounting for age, education, BMI and vascular comorbidities. Sleep efficiency further correlated positively with cognitive preservation, suggesting restorative sleep quality as a protective factor. Importantly, individuals who improved their sleep duration during follow-up experienced attenuated cognitive decline, nearly comparable to those consistently sleeping 6-8 hours. Finally, depressive symptoms increased disproportionately among chronically sleep-deprived participants, potentially amplifying adverse effects on cognition.

Table 1 (see PDF) shows Participants were evenly distributed by sex and age across sleep duration groups, but short sleepers had higher rates of comorbid conditions. Table 2 (see PDF) shows short sleepers had significantly lower baseline subjective sleep quality scores. Table 3 (see PDF) shows Global cognitive scores declined most in the <6 hrs group over the 3-year period. Table 4 (see PDF) shows Memory scores showed the steepest decline in short sleepers. Table 5 (see PDF) shows Executive function declined significantly more in short sleepers. Table 6 (see PDF) shows Attention and working memory performance deteriorated more in sleep-deprived individuals. Table 7 (see PDF) shows Sleep duration was an independent predictor of cognitive decline after adjusting for confounders. Table 8 (see PDF) shows higher sleep efficiency was positively associated with cognitive preservation. Table 9 (see PDF) shows Individuals who improved their sleep duration showed attenuated cognitive decline. Table 10 (see PDF) shows short sleepers also had greater increases in depressive symptoms, possibly compounding cognitive effects.

## Discussion:

This prospective study demonstrates a significant association between chronic sleep deprivation and accelerated cognitive decline in middle-aged adults over a 3-year period. Participants who consistently slept less than six hours per night experienced greater reductions in global cognitive function, particularly in domains of memory, attention and executive function. These findings held true even after controlling for potential confounders such as age, BMI, education, depression and comorbid conditions like hypertension and diabetes, underscoring the independent role of insufficient sleep in neurocognitive deterioration [11]. The biological plausibility of this association is supported by multiple mechanisms. Sleep is crucial for synaptic pruning, memory consolidation and clearance of neurotoxic waste through the glymphatic system. Short sleep duration disrupts these restorative processes, potentially leading to neuroinflammation and accumulation of beta-amyloid and tau proteins-early markers of neurodegeneration [12]. Elevated morning cortisol levels and increased depressive symptoms among short sleepers in this cohort further suggest that chronic stress and mood disturbances may act as intermediaries exacerbating cognitive decline. Moreover, individuals who improved their sleep duration during the study period exhibited a slower rate of cognitive deterioration, indicating that sleep may be a modifiable risk factor with preventive potential [13]. The additive negative impact of sleep deprivation and hypertension on memory suggests that coexisting vascular and sleep-related risks can synergistically impair brain function. Importantly, while long sleepers (>8 hours) showed slightly more cognitive decline than normal sleepers, their changes were significantly less severe than those of the short sleep group, pointing toward a U-shaped relationship that warrants further investigation. Given the growing prevalence of sleep deprivation due to modern lifestyle pressures, these findings emphasize the importance of integrating sleep hygiene into midlife health promotion strategies. Future longitudinal studies with neuroimaging and biomarker integration may offer deeper insights into the sleep-cognition pathway and help shape early interventions for cognitive preservation [14].

## Conclusion:

Chronic sleep deprivation is a significant, independent predictor of cognitive decline among middle-aged adults, particularly affecting memory, executive function and attention. Individuals sleeping less than six hours per night over multiple years demonstrated accelerated cognitive deterioration, even after adjusting for age, education, comorbidities and mental health factors. Thus, we show the critical role of sufficient and high-quality sleep in maintaining cognitive health during midlife. Addressing sleep habits through early lifestyle interventions may offer a promising and accessible strategy to delay or prevent the onset of cognitive impairment.
